# Supplementary material for: Low-Pb High-Piezoelectric Ceramic System (1−x)Ba(Zr0.18Ti0.82)O3–x(Ba0.78Pb0.22)TiO3
Source: Materials (Basel). 2022 Jul 7;15(14):4760. doi: 10.3390/ma15144760 (PMC9322182; doi:10.3390/ma15144760)
Supplement: Supplementary file 1 [file materials-15-04760-s001.zip › materials-1749700-supplementary.pdf]

Supplementary

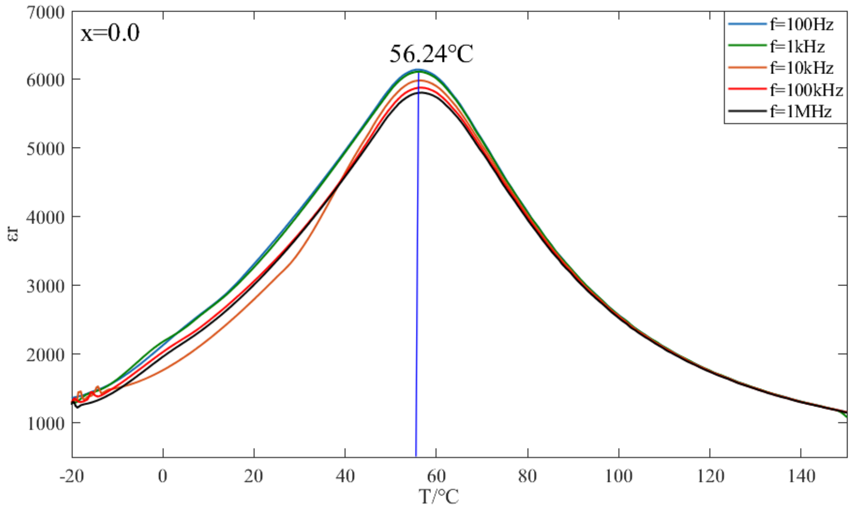

Figure S1. Dielectric permittivity versus temperature curve (100 Hz, 1 kHz, 10 kHz, 100 kHz, 1 MHz) of x=0.

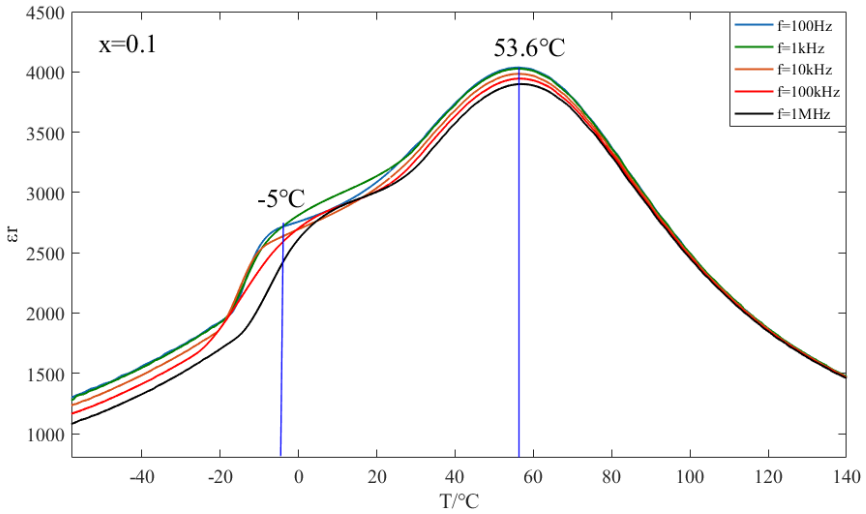

Figure S2. Dielectric permittivity versus temperature curve (100 Hz, 1 kHz, 10 kHz, 100 kHz, 1 MHz) of x=0.1.

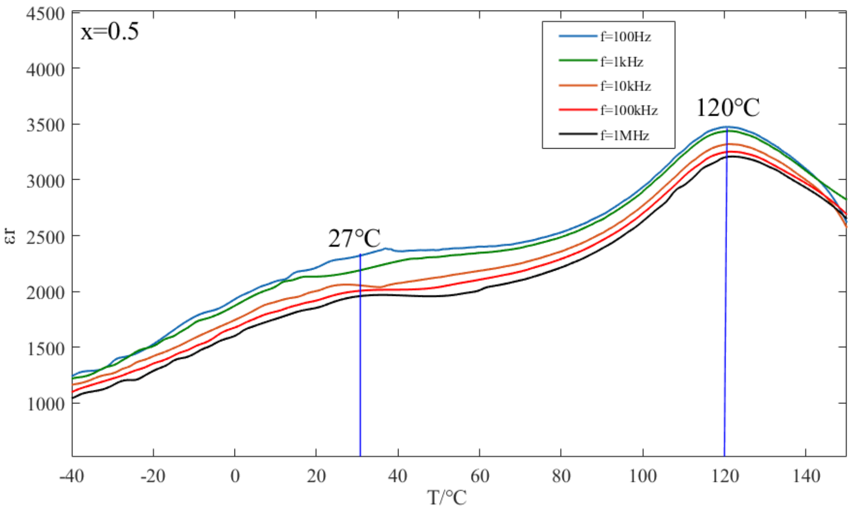

Figure S3. Dielectric permittivity versus temperature curve (100 Hz, 1 kHz, 10 kHz, 100 kHz, 1 MHz) of x=0.5.

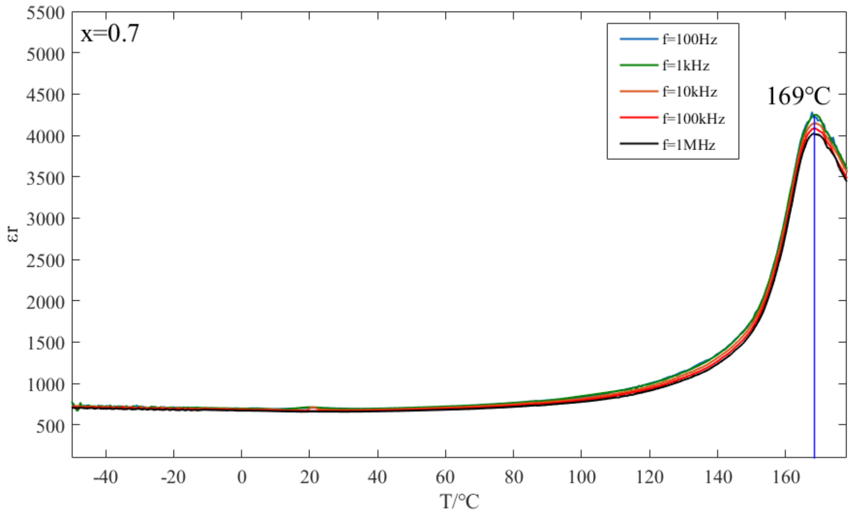

Figure S4. Dielectric permittivity versus temperature curve (100 Hz, 1 kHz, 10 kHz, 100 kHz, 1 MHz) of x=0.7.
